# Anti-Obesity and Metabolic Effects of Forskolin in Obese C57BL/6J Mice

**DOI:** 10.3390/ijms26146607

**Published:** 2025-07-10

**Authors:** Mehrnaz Abbasi, Fang Zhou, Ngoc Kim Ly, Austin Taylor, Qiaobin Hu, Jinhua Chi, Haiwei Gu, Shu Wang

**Affiliations:** 1 College of Health Solutions, Arizona State University, Phoenix, AZ 85004, USA; mza0264@auburn.edu (M.A.); ajtayl25@asu.edu (A.T.); qiaobinchris@gmail.com (Q.H.); 2 College of Human Sciences, Auburn University, Auburn, AL 36830, USA

**Keywords:** forskolin, white adipose tissue browning, obesity, metabolic health, subcutaneous WAT delivery

## Abstract

Forskolin (FSK) induces the browning of white adipose tissue (WAT) through the activation of adenylate cyclase (AC) and cyclic adenosine monophosphate (cAMP) generation. When administered intravenously or orally, FSK undergoes significant metabolism and accumulation in the liver and other tissues, resulting in high side effects and low anti-obesity effects due to trivial amounts reaching WAT. This study examines the potential anti-obesity and metabolic effects of the inguinal WAT (IWAT) delivery of FSK in high-fat diet-induced C57BL/6J obese mice. Mice received one of the following treatments twice weekly for 4 weeks: 1. Control into both IWAT depots (Con^both^); 2. FSK 15 mg/kg body weight (BW)/injection into both inguinal WAT (IWAT) depots (FSK15^both^); 3. FSK 7.5 mg/kg BW/injection into both IWAT depots (FSK7.5^both^); and 4. FSK 7.5 mg/kg BW/injection into the left IWAT depot (FSK7.5^left^). Both the FSK15^both^ and FSK7.5^both^ treatments improved metabolic parameters by lowering blood glucose, enhancing glucose tolerance, and reducing serum insulin and cholesterol. The FSK15^both^ treatment had a greater impact on IWAT, resulting in smaller adipocytes and increased expression of Ucp1 and Tmem26 mRNA levels. All FSK treatments also reduced inflammatory and lipogenic markers in the liver, indicating improved hepatic metabolism. These findings suggest that local delivery of FSK into subcutaneous WAT is a potential strategy for combating obesity and improving metabolic health. However, further studies are needed to confirm the statistical and biological significance of these effects.

## 1. Introduction

Forskolin (FSK, C22H34O7) is a natural labdane diterpene found in the roots of Coleus forskohlii [1,2]. FSK activates adenylyl cyclase (AC), increasing cell cAMP levels [3]. FSK is also used to induce adipocyte browning in mice [4,5]. By activating AC and increasing cAMP levels, FSK triggers downstream pathways involving protein kinase A (PKA), cAMP response element-binding protein (CRBP), p38 mitogen-activated protein kinase (MAPK), adipose triglyceride lipase (ATGL), and hormone-sensitive lipase (HSL) [5,6,7,8]. These cascades promote the expression of uncoupling protein 1 (*Ucp1*), peroxisome proliferator-activated receptor γ co-activator1α (*Pgc1α*), and cell death-inducing DFFA-like effector a (*Cidea*), induce browning of WAT, enhance lipolysis, and reduce fat storage. Activation of brown and beige adipocytes increases substrate oxidation and energy expenditure while improving metabolic health. However, the precise metabolism of FSK and the FSK-induced browning mechanism in vivo, particularly through direct delivery of FSK to IWAT (a type of subcutaneous WAT in mice), remains unknown, creating a significant gap in understanding.

Previous studies indicate that FSK has an anti-obesity effect [4,9,10], but its browning function is inconclusive. The limitations of FSK, such as low stability, poor aqueous solubility (around 0.01 mg/mL), low bioavailability (<1%) [11,12], and a lack of targeting specificity, likely contribute to these uncertainties. When administered intravenously or orally, FSK undergoes significant metabolism and accumulation in the liver and other tissues, resulting in trivial amounts reaching WAT. High FSK doses (>30 mg/kg body weight) may lead to hepatic toxicity, increased heart rate, and decreased blood pressure [11,12,13]. Since subcutaneous WAT is more accessible and responsive to browning agents than other types of WAT [14,15], the local delivery of FSK to subcutaneous WAT (IWAT) holds promise for developing an effective and safe treatment for obesity.

This study examines the anti-obesity and metabolic effects of IWAT delivery of FSK in high-fat diet (HFD)-induced obese mice. We hypothesize that the browning strategy of using the IWAT delivery of FSK might be a potential breakthrough for combating obesity and its comorbidities in a more efficient and safe approach.

## 2. Results

### 2.1. The Effects of FSK on Body Weight and Body Fat in HFD-Induced Obese Mice

**Figure 1:**
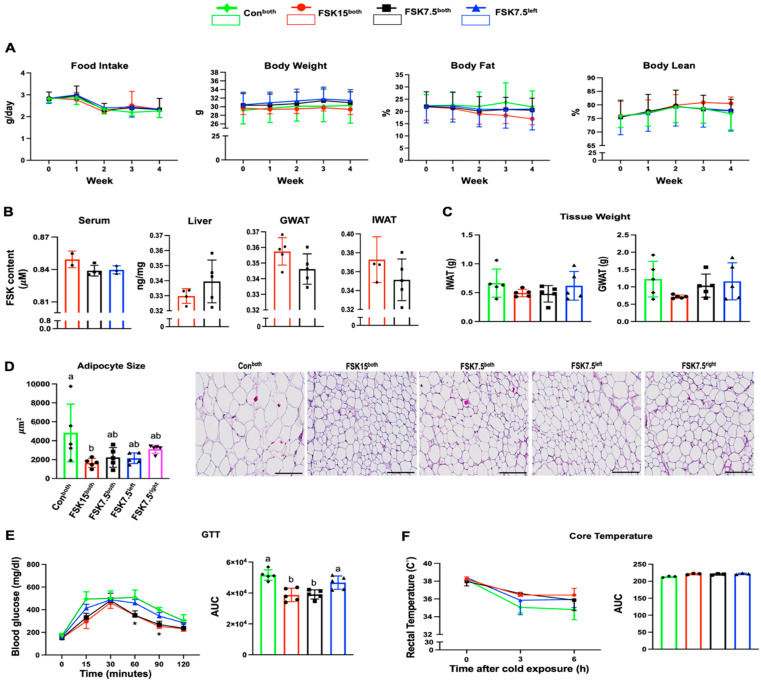
Anti-obesity and metabolic effects of FSK on high-fat diet-induced obese C57BL/6J. (**A**) Food intake, body weight, fat%, and lean%. (**B**) FSK content in serum, liver, GWAT, and IWAT. (**C**) Tissue weight IWAT and GWAT. (**D**) Adipocyte size and representative H&E histological images of IWAT (scale bar: 200 µm). (**E**) Blood glucose level and glucose tolerance test area under the curve (AUC). (**F**) Core body temperature and AUC. (*) Lower blood glucose. Values are mean ± SEM, *n* = 2–5 per treatment. Bars without a common superscript differ, *p* < 0.05. Bars or lines without a common superscript differ, *p* < 0.05 by one-way ANOVA followed by Tukey HSD Post Hoc test.

There were no significant differences in food intake among all treatment groups. FSK15^both^-treated mice had the lowest body weight, but the differences did not reach statistical significance. Compared to Con^both^ mice, FSK7.5^left^, FSK7.5^both^, and FSK15^both^ had a 1%, 1%, and 5% lower body fat percent (body fat%), respectively. Since FSK15^both^-treated mice had the lowest body fat%, they had the highest lean body percentage (lean body%) among all treatment groups (Figure 1A). The differences were not statistically significant. There were no significant differences in serum, liver, gonadal WAT (GWAT), and IWAT FSK content among the FSK15^both^, FSK7.5^both^, and FSK7.5^left^ groups (Figure 1B).

### 2.2. The Effects of FSK on IWAT and GWAT Weights

FSK15^both^- and FSK7.5^both^-treated mice had 1.34- and 1.38-fold lower IWAT weights than the Con^both^ treatment groups. FSK15^both^-treated mice had 1.6- and 1.7-fold lower GWAT weights than mice in the FSK7.5^left^- and Con^both^ treatment groups, respectively. The differences were not statistically significant (Figure 1C).

### 2.3. The Effects of FSK on Adipocyte Size in IWAT

FSK15^both^-treated mice had the smallest average adipocyte size in IWAT among all treatment groups. The average adipocyte size in IWAT was 2.9- (*p* = 0.023), 2.15-, and 2.24-fold lower in the FSK15^both^-, FSK7.5^both^-, and FSK7.5^left^-treated mice than in the Con^both^-treated mice (Figure 1D).

### 2.4. The Effects of FSK on Blood Glucose Levels and GTT-AUC

GTT-AUC in FSK15^both^- and FSK7.5^both^-treated mice were significantly lower than mice in the FSK7.5^left^ (1.2- and 1.19-fold, *p* < 0.05) and Con^both^ (1.33- and 1.32-fold, *p* < 0.001) treatment groups (Figure 1E).

### 2.5. The Effects of FSK on Body Core Temperature

After 6 h of cold challenge at 4 °C, the body temperature of mice in the Con^both^ groups dropped to ~34.8 °C. However, the body temperatures of mice in the FSK7.5^both^-, FSK7.5^left^-, and FSK15^both^-treated mice were about 36.0 °C, 36.0 °C, and 36.4 °C. Although mice in the FSK treatment groups maintained their body temperature more efficiently during the cold tolerance test, the differences were not statistically significant (Figure 1F).

### 2.6. The Effects of FSK on Energy Metabolism

**Figure 2:**
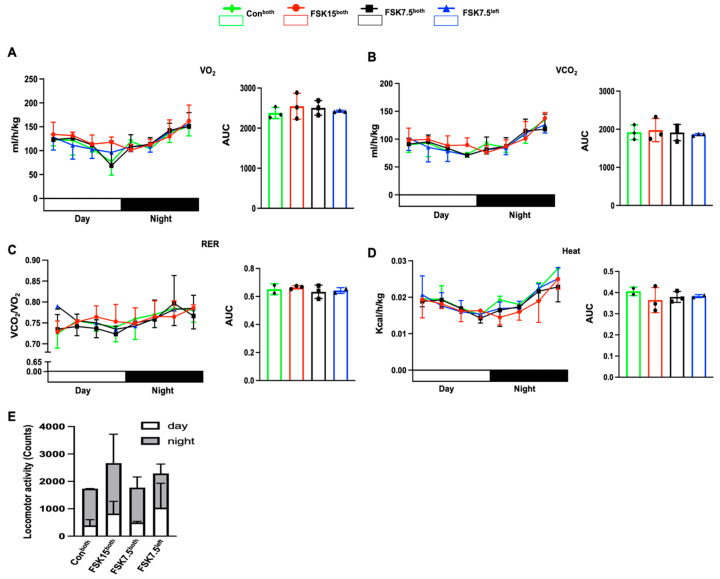
Indirect calorimetric analysis of high-fat diet (HFD)-induced obese C57BL/6J mice. (**A**) O_2_ consumption (VO_2_) and the area under the curve (AUC). (**B**) CO_2_ production (VCO_2_) and AUC (VO_2_ and VCO_2_ normalized to lean body mass). (**C**) Respiratory exchange ratio (RER) and AUC. (**D**) Heat and AUC. (**E**) Locomotor activity. Values are mean ± SEM, *n* = 3 per treatment.

Among all treatment groups, FSK15^both^-treated mice had the highest oxygen consumption, expressed by VO_2_ [mL/h/kg body weight]; CO_2_ production, expressed by VCO_2_ [mL/h/kg body weight]; respiration exchange ratio (RER), reflected by released CO_2_/consumed O_2_; and locomotor activity [count]; however, the differences are not statistically significant (Figure 2A–E).

### 2.7. The Effects of FSK on Serum Insulin and Total Cholesterol Levels

**Figure 3:**
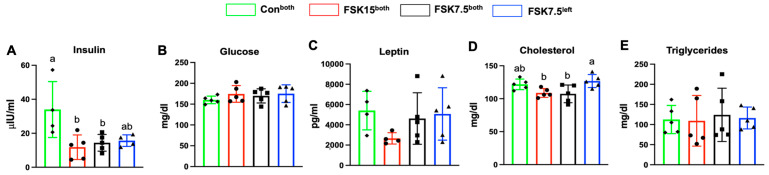
Serum biochemistry parameters analysis in high-fat diet (HFD)-induced obese C57BL/6J mice. (**A**) Insulin, (**B**) glucose, (**C**) leptin, (**D**) cholesterol, and (**E**) triglycerides. Values are mean ± SEM *n* = 5 per treatment. Bars without a common superscript differ, *p* < 0.05, by one-way ANOVA followed by Tukey HSD Post Hoc test.

FSK15^both^- and FSK7.5^both^-treated mice had 2.87- and 2.17-fold lower serum insulin levels than Con^both^-treated mice (*p* < 0.05). FSK15^both^-treated mice had 1.9- and 2-fold lower serum leptin levels than mice in the FSK7.5^left^ and Con^both^ treatment groups; however, the difference was not statistically significant. FSK15^both^- and FSK7.5^both^-treated mice had 1.16- and 1.18-fold lower serum total cholesterol levels than mice in the FSK7.5^left^-treated mice (*p* < 0.05). Differences in glucose and triglyceride levels were not significant among treatment groups (Figure 3A–E). 

### 2.8. The Effects of FSK on the Expression of Browning Markers in IWAT

**Figure 4:**
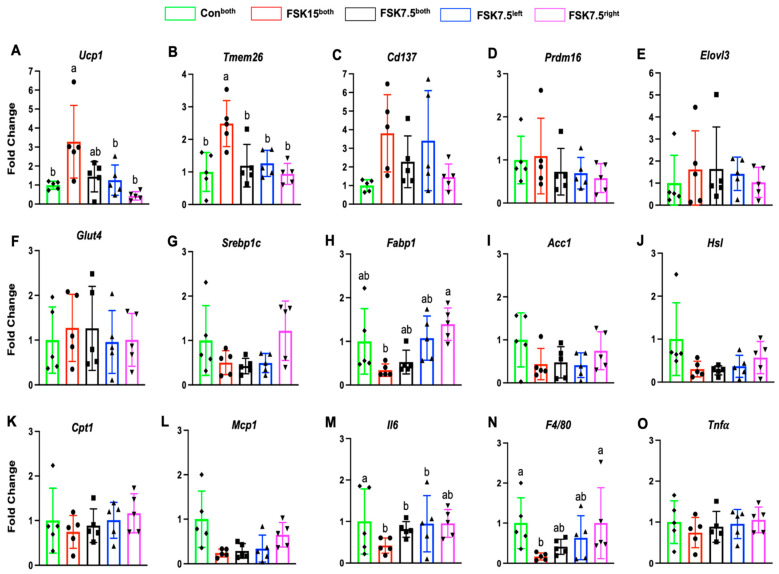
IWAT mRNA levels of browning, glucogenic, lipogenic, and inflammatory markers. Browning markers; (**A**) Ucp1, (**B**) Tmem26, (**C**) Cd137, (**D**) Prdm16, and (**E**) Elovl3, (**F**) Glut4, Lipogenic markers; (**G**) Srebp1c, (**H**) Fabp1, (**I**) Acc1, (**J**) Hsl, and (**K**) Cpt1, Inflammatory markers; (**L**) Mcp1, (**M**) Il6, (**N**) F4/80, and (**O**) Tnfα. Values are mean ± SEM *n* = 5 per treatment. Bars without a common superscript differ, *p* < 0.05 by one-way ANOVA followed by Tukey HSD Post Hoc test.

**Figure 5:**
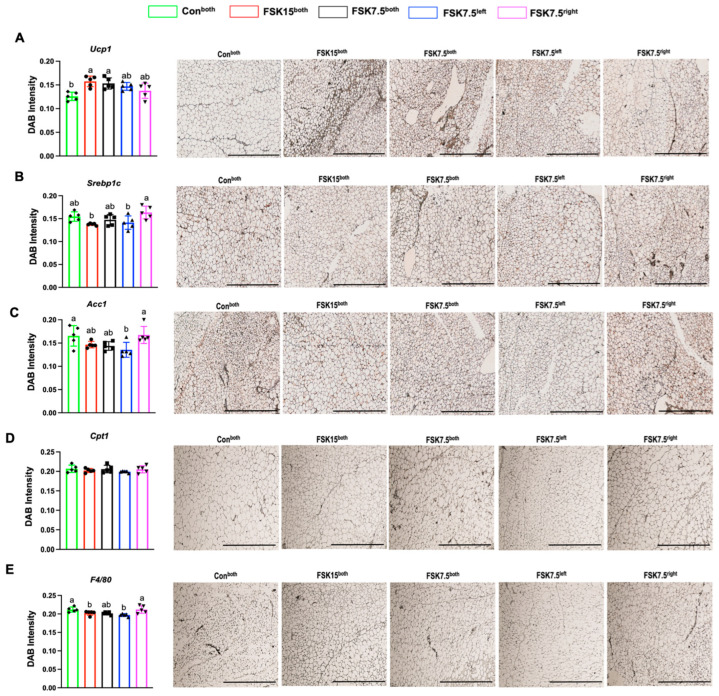
IWAT protein expression levels of browning, lipogenic, and inflammatory markers. Reciprocal intensity of the chromogen stain and representative immunohistochemistry staining images of IWAT (Scale bar: 200 µm): (**A**) Ucp1, (**B**) Srebp1c, (**C**) Acc1, (**D**) Cpt1, and (**E**) F4/80. Values are mean ± SEM *n* = 5 per treatment. Bars without a common superscript differ, *p* < 0.05 by one-way ANOVA followed by Tukey HSD Post Hoc test.

FSK15^both^-, FSK7.5^both^-, and FSK7.5^left^-treated mice had higher IWAT mRNA levels of Ucp1 (3.27- (*p* = 0.003), 1.43-, and 1.25-fold), transmembrane protein 26 (Tmem26, 2.48- (*p* = 0.002), 1.19-, and 1.26-fold), and Cd137 (3.8-, 2.27-, and 3.4-fold) (*p* = 0.067) than Con^both^-treated mice (Figure 4A–C). Differences in the IWAT mRNA levels of Prdm16 and Elvol3 were not significant among treatment groups (Figure 4D,E). IWAT UCP1 protein expression levels were 1.24- and 1.21-fold (*p* < 0.02) higher in FSK15^both^- and FSK7.5^both^-treated mice as compared to Con^both^-treated mice (Figure 5A).

### 2.9. The Effects of FSK on the Expression of Glut4 Adipogenic Markers in IWAT

IWAT mRNA levels of glucose transporter 4 (Glut4, 1.27- and 1.26-fold) were higher in FSK15^both^- and FSK7.5^both^-treated mice than Con^both^-treated mice (Figure 4F). However, the difference was not statistically significant.

As compared to Con^both^-treated mice, FSK15^both^-, FSK7.5^both^-, and FSK7.5^left^-treated mice had lower IWAT mRNA levels of adipogenic markers; sterol regulatory element-binding transcription factor 1 (Srebp1c, 1.99-, 2.35-, and 2.02-fold) (*p* = 0.063), fatty acid-binding protein 1 (Fabp1, 1.76-, 1.89-, and 0.93-fold), acetyl-CoA-carboxylase 1 (Acc1, 2.28-, 2.09-, and 2.43-fold), hormone-sensitive lipase (Hsl, 3.28-, 3.64-, and 2.65-fold), and Cpt1 (1.33-, 1.12-, and 0.99-fold) (Figure 4G–K).

As compared to Con^both^-treated mice, FSK15^both^-, FSK7.5^both^-, and FSK7.5^left^-treated mice had lower IWAT protein expression levels of SREBP1c (1.12-, 1.05-, and 1.09-fold), ACC1 (1.12-, 1.15-, and 1.22-fold (*p* < 0.05)), and CPT1 (1.02-, 1.00-, and 1.03-fold) (Figure 5B–D).

### 2.10. The Effects of FSK on the Expression of Inflammatory Markers in IWAT

As compared to Con^both^-treated mice, FSK15^both^-, FSK7.5^both^-, and FSK7.5^left^-treated mice had lower IWAT mRNA levels of inflammatory markers; monocyte chemoattractant protein (Mcp1, 3.23-, 1.68-, and 1.44-fold), Interleukin-6 (Il6, 4.12-, 3.46-, and 2.92-fold) (*p* < 0.05), EGF-like module-containing mucin-like hormone receptor-like 1 (F4/80, 3.35- (*p* < 0.05), 3.23-, and 2.16-fold), and tumor necrosis factor α (Tnfα, 1.33-, 1.12-, and 1.04-fold) (Figure 4L–O).

IWAT protein expression levels of F4/80 (1.04- and 1.07-fold) (*p* < 0.05) were lower in FSK15^both^- and FSK7.5^left^-treated mice than mice in Con^both^-treated mice (Figure 5E).

### 2.11. The Effects of FSK on the Expression of Ac1 in IWAT

**Figure 6:**
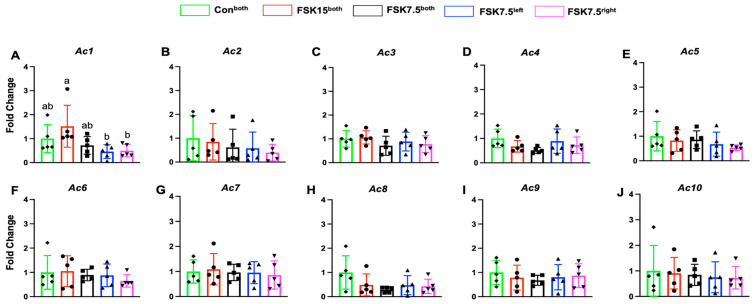
IWAT mRNA levels of adenylate cyclase 1–10. (**A**–**E**) Ac1–5; (**F**–**J**) Ac6–10. Values are mean ± SEM *n* = 5 per treatment. Bars without a common superscript differ, *p* < 0.05 by one-way ANOVA followed by Tukey HSD Post Hoc test.

As compared to FSK7.5^left^- and FSK7.5^right^-treated mice, FSK15^both^-treated mice had higher IWAT mRNA levels of Ac1 (3.26- and 3.01-fold) (*p* < 0.05). There were no significant differences in Ac2–10 expression in IWAT among all groups (Figure 6A–J).

### 2.12. The Effects of FSK on the Expression of Glut2 and Adipogenic Markers in the Liver

**Figure 7:**
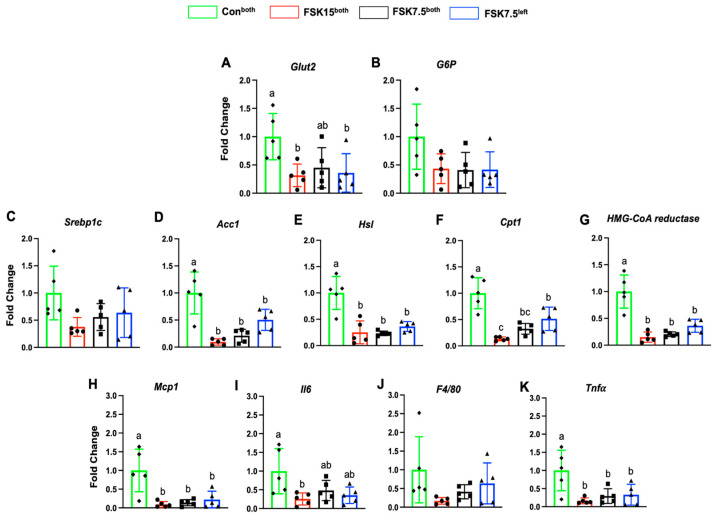
Liver mRNA levels of glucogenic, lipogenic, and inflammatory markers. Glucogenic markers; (**A**) Glut2 and (**B**) G6P, Lipogenic markers; (**C**) Srebp1c, (**D**) Acc1, (**E**) Hsl, (**F**) Cpt1, (**G**) HMG-CoA reductase Inflammatory markers; (**H**) Mcp1, (**I**) Il6, (**J**) F4/80, and (**K**) Tnfα. Values are mean ± SEM *n* = 5 per treatment. Bars without a common superscript differ, *p* < 0.05, by one-way ANOVA followed by Tukey HSD Post Hoc test.

As compared to Con^both^-treated mice, FSK15^both^- and FSK7.5^left^-treated mice had lower liver mRNA levels of glucose transporter 2 (Glut2, 3.16- and 2.69-fold) (*p* < 0.05) and glucose 6 phosphatase (G6P, 2.3- and 2.39-fold) (*p* = 0.07) (Figure 7A,B).

As compared to Con^both^-treated mice, FSK15^both^-, FSK7.5^both^-, and FSK7.5^left^-treated mice had lower liver mRNA levels of Srebp1c (2.65-, 1.79-, and 1.56-fold) (*p* = 0.09), Acc1 (10.5-, 4.74-, and 1.98-fold) (*p* < 0.02), Hsl (3.97-, 4.29-, and 2.76-fold) (*p* < 0.001), Cpt1 (7.52-, 3.08-, and 1.94-fold) (*p* < 0.01), and 3-hydroxy-3-methyl-glutaryl-coenzyme A reductase (HMG-CoA reductase, 6.62-, 4.87-, and 2.76-fold) (*p* < 0.001) (Figure 7C–G).

**Figure 8:**
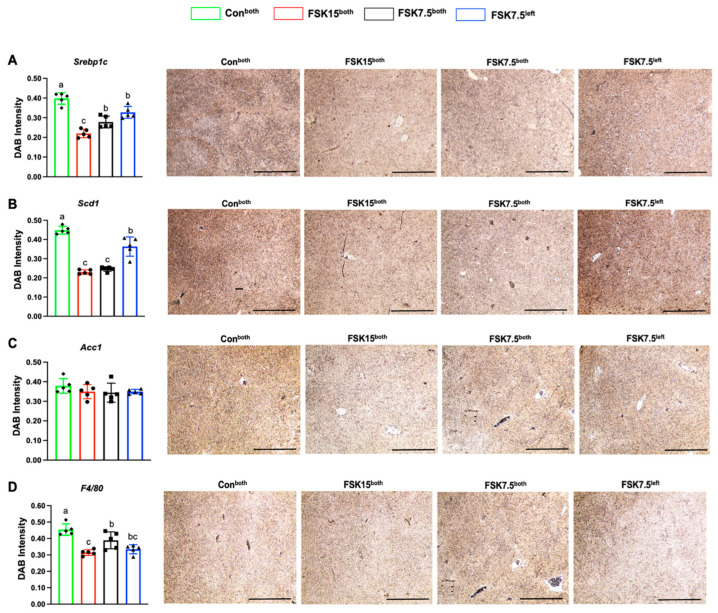
Liver protein expression levels of lipogenic and inflammatory markers. Reciprocal intensity of the chromogen stain and representative immunohistochemistry staining images of IWAT (Scale bar: 200 µm): (**A**) Srebp1c, (**B**) Scd1, (**C**) Acc1, and (**D**) F4/80. Values are mean ± SEM *n* = 5 per treatment. Bars without a common superscript differ, *p* < 0.05, by one-way ANOVA followed by Tukey HSD Post Hoc test.

Liver protein expression levels of SREBP1c (1.81-, 1.43-, and 1.21-fold) (*p* < 0.001), SCD1 (1.94-, 1.81-, and 1.23-fold) (*p* < 0.001), and ACC1 (1.08-, 1.09-, and 1.08-fold) were lower in FSK15^both^-, FSK7.5^both^-, and FSK7.5^left^-treated mice than Con^both^-treated mice (Figure 8A–C).

### 2.13. The Effects of FSK on the Expression of Inflammatory Markers in the Liver

As compared to Con^both^-treated mice, FSK15^both^-, FSK7.5^both^-, and FSK7.5^left^-treated mice had lower liver mRNA levels of inflammatory markers; Mcp1 (10.84-, 7.21-, and 4.53-fold) (*p* < 0.001), Il6 (3.91- (*p* < 0.05), 2.07-, and 2.82-fold), F4/80 (5.89-, 2.42-, and 1.57-fold), and Tnfα (6.15-, 3.40-, and 3.00-fold) (*p* = 0.005) (Figure 7H–K).

Liver protein expression levels of F4/80 (1.44-, 1.16-, and 1.35-fold) (*p* < 0.001) were lower in FSK15^both^-, FSK7.5^both^-, and FSK7.5^left^-treated mice than Con^both^-treated mice (Figure 8D).

### 2.14. The Effects of FSK and Tween80 on Renal, Liver, or Electrolyte Safety Parameters

No significant differences among the four groups were found in the serum chemistry, including renal function, liver function, and electrolytes (Supplementary Table S1).

## 3. Discussion

Administration of FSK to IWAT in obese mice was associated with trends toward reduced body weight and body fat, though these differences did not reach statistical significance. Mice receiving FSK also showed trends toward a lower WAT mass and reduced adipocyte size and demonstrated improved glucose tolerance and lower serum insulin and cholesterol levels compared to the controls. Furthermore, the FSK-treated group exhibited a higher expression of genes related to adipose tissue browning and thermogenesis in IWAT, with increased Ucp1 protein levels as well. Overall, the results suggest potential metabolic benefits of FSK, including favorable effects on glucose metabolism, insulin sensitivity, inflammation and lipid regulation, and WAT browning; however, these findings should be interpreted with caution, as the observed changes in body weight, fat mass, and other metabolic parameters were not consistently significant.

Studies have found that oral FSK supplementation can reduce the body fat percentage and increase lean body mass in overweight and obese individuals without significantly affecting body weight. FSK has been administered orally as ForseLean (250 mg of 10% FSK extract twice daily) for 8 or 12 weeks [9,16]; however, these studies did not assess or report forskolin’s potential to induce adipose tissue browning.

In most animal studies, FSK was given as a dietary supplement from Coleus for-skohlii root extract (CFE), leading to lower food intake, body weight, and visceral fat, but with higher liver weights in mice. Although liver enzyme activities increased with CFE intake, pure FSK alone did not alter liver responses, suggesting it is likely not harmful [13]. In another study, male ICR mice given 0.3 or 1% FSK from CFE for 2 weeks showed increased liver weight, plasma AST/ALT/ALP levels, and liver lipogenic gene expression, suggesting enhanced de novo lipogenesis with CFE treatment [17]. However, no significant change in liver lipogenic genes was observed in the current study, potentially due to differences in administration route (oral vs. subcutaneous), doses, and animal models used. 

In a study on obese male mice, FSK administration showed a gradual reduction in body weight at 8 weeks, but no significant differences in food intake, serum lipids, or glucose tolerance test [4]; however, subcutaneous adipocyte diameter was reduced. In vitro, FSK decreased adipocyte differentiation, triglyceride content, and Glut4 expression in cells [4]. Similarly, our study showed a decreasing trend in the body weight and fat percentage with FSK treatment, although not statistically significant, potentially due to differences in dosage, duration, and delivery route. 

FSK can activate all human adenylate cyclase (AC) isoforms except AC9 in spermatozoa [18,19]. Different AC isoforms have varying sensitivities to FSK, with the highest potency at AC1 and the lowest at AC2 [20]. Consistently, our study found higher AC1 expression in the IWAT of FSK-treated mice compared to other groups. The ability of FSK to elevate cAMP levels exerts significant physiological effects on various biochemical processes in the body. 

In our study, FSK administration decreased IWAT and GWAT weights and adipocyte size in IWAT. FSK’s lipolytic potential has been demonstrated in vitro, potentially reducing adipocyte size and improving energy/glucose/lipid metabolism by increasing lipolysis and decreasing adipocyte differentiation [4,21]. FSK increases cAMP levels in adipose tissue, activating PKA, which phosphorylates perilipin A and facilitates HSL translocation to lipid droplets. Activated HSL breaks down triglycerides, releasing free fatty acids and contributing to lower fat mass through triglyceride hydrolysis in adipocytes [19,21].

The hallmark of WAT browning is the formation of brown-like “beige” adipocytes with increased mitochondria and Ucp1 expression, enhancing thermogenesis and energy expenditure. Increased cAMP levels and subsequent PKA activation may contribute to this browning process [22]. Our study found increased expression of browning markers Ucp1 and TMEM26 in the IWAT of forskolin-treated mice, suggesting that FSK promoted the browning of WAT, which could improve the metabolic profile and reduce body weight. Similarly, treatment of differentiated human adipocytes with FSK-loaded lipid-coated mesoporous silica nanoparticles (LCMSN) increased Ucp1 expression, a key thermogenic marker, with significant upregulation observed in as early as 3 hours, and a 40-fold increase after 6 hours of incubation. LCMSN-FSK also significantly enhanced lipolysis and metabolic activity, as indicated by the increased oxygen consumption rate. These effects were observed across a range of FSK doses (1–50 μg), with the highest tested dose in vivo (HFD-induced obese C57BL/6J mice, IAWT injection) being 50 μg/mouse initially, and 500 μg/mouse for extended treatment [23].

We found decreased expression of hepatic lipogenic markers with FSK treatment. The cAMP/PKA signaling pathway plays a crucial role in regulating hepatic carbohydrate and lipid metabolism by modulating cAMP-responsive transcription factors like CREB. Activated CREB represses expression of lipogenic genes, including by downregulating PPAR-γ, a key regulator of lipogenesis, and Srebp1c, which controls fatty acid synthase and other lipogenic enzymes. This highlights the cAMP/CREB pathway’s significant role in regulating hepatic lipid metabolism and the potential mechanism by which FSK reduces hepatic lipogenesis [24,25]. 

Our study found decreased Glut4 and Glut2 expression in IWAT and the liver, respectively, with FSK treatment. Elevated cAMP levels promote glucose production by in-creasing gluconeogenic enzyme transcription [3]. FSK stimulates insulin release from pancreatic beta cells by raising cAMP, but insulin subsequently suppresses cAMP in the liver and adipose tissue, coordinating glucose and lipid metabolism. Previous studies suggest that FSK inhibits glucose transporters like Glut4, possibly by stimulating cAMP accumulation or altering glucose metabolism independent of cAMP. The interplay between cAMP, insulin, and glucose/lipid metabolism in different tissues may contribute to FSK’s metabolic effects [3,4,26]. 

In our study, we found a reduced expression of inflammatory markers in both the IWAT and liver, possibly due to decreased fat storage. Previous research suggests that FSK has anti-inflammatory effects by increasing cAMP levels, PKA activity, CREB phosphorylation, and CREB interaction with CBP in adipocytes [27,28,29]. 

FSK and isoforskolin have been shown to reduce inflammation induced by lipopolysaccharide in leukocytes, macrophages, and dendritic cells by downregulating cytokines like IL-1β, TNF-α, and IL-6, potentially involving the TLR4/MyD88/NF-κB pathway [30]. The cAMP/CREB pathway can also suppress the hepatic expression of inflammatory genes like Tnfα and Il10 [31,32]. 

While our results suggest promising potential for the anti-obesity and metabolic benefits of FSK, it is important to recognize that the majority of the findings did not reach statistical significance, likely due to the relatively small sample size. Therefore, these results should be interpreted as preliminary. Further studies with larger sample sizes are needed to confirm the observed effects.

## 4. Materials and Methods

### 4.1. Materials

FSK was purchased from Cayman Chemical Co. (Ann Arbor, MI, USA). Anti-*Ucp1*, sterol regulatory element-binding protein-1c (*Serbp1c*), Acetyl-CoA carboxylase 1 (*Acc1*), stearoyl-CoA desaturase 1 (*Scd1*), Carnitine palmitoyltransferase I (*Cpt1*), and EGF-like module-containing mucin-like hormone receptor-like 1 (*F4/80*) antibodies and primers were purchased from Sigma-Aldrich, Inc. (St. Louis, MO, USA). TRIzol^®^ reagent, Maxima First Strand cDNA Synthesis Kit, insulin and leptin ELISA kits, PowerUp SYBR™ Green Master Mix, and TWEEN^®^ 80 were obtained from Thermo Fisher Scientific (Pittsburgh, PA, USA). Vectastain ABC kit and Vector Hematoxylin were purchased from Vector Laboratories (Burlingame, CA, USA).

### 4.2. Animals

Male C57BL/6J mice from Jackson Laboratory at 6 weeks of age were housed at 22–24 °C, 45% relative humidity, a daily 12-h light/dark cycle, and had free access to water and diet. After 1-week acclimation, mice were fed HFD for 8 weeks (45% energy from fat, D12451, Research Diets, Inc., New Brunswick, NJ, USA) to induce obesity. After 4 weeks, mice were weighed and randomly assigned into one of the following groups (*n* = 5/treatment group): 1. Tween 80 Control into both IWAT depots (Con^both^); 2. FSK 15 mg/kg Body Weight (BW)/injection into both inguinal WAT (IWAT) depots (FSK15^both^); 3. FSK 7.5 mg/kg BW/injection into both IWAT depots (FSK7.5^both^); 4. FSK 7.5 mg/kg BW/injection into the left IWAT depot (FSK7.5^left^). FSK (dissolved in Tween 80 at a 1:50 mass ratio) or Tween 80 Control were injected into IWAT twice weekly for 4 weeks while mice were fed on the HFD. In FSK7.5^left^, FSK was only injected in the left side of IWAT, and FSK7.5^right^ was considered its self-control. A glucose tolerance test (GTT), indirect calorimetry, and cold tolerance test were conducted in the third and fourth weeks of treatment. Mice were euthanized at the end of the fourth week, and blood and tissue samples were collected for analysis.

### 4.3. Body Composition

Mouse body composition was determined weekly using nuclear magnetic resonance technology by EchoMRI™ (EchoMRI LLC, Houston, TX, USA).

### 4.4. GTT

For the GTT, mice fast for 6 h, then are intraperitoneally injected with glucose (2 g/kg BW). Blood is collected from the tail vein at 0, 15, 30, 60, 90, and 120 min post-injection, and glucose levels are measured using a glucometer. Blood glucose concentrations are plotted against time, and the area under the curve (AUC) is calculated using the trapezoidal rule formula, which is as follows: AUC = ((C1 + C2)/2) × (T2 − T1), where C1 and C2 are consecutive glucose concentrations, and T1 and T2 are the corresponding time points.

### 4.5. Energy Expenditure

Metabolic parameters such as O_2_ consumption (VO_2_ [mL/h/kg]), CO_2_ production (VCO_2_ [mL/h/kg]), respiratory exchange ratio (RER), and energy expenditure (H1 [kcal/h/kg]) were measured by indirect calorimetry using the following Comprehensive Lab Animal Monitoring System: Oxymax-CLAMS-HC (Columbus Instruments, Columbus, OH, USA). The volume of O_2_ consumption (VO_2_) and the volume of CO_2_ production (VCO_2_) were normalized to body lean mass.

### 4.6. Cold-Tolerance Test

Before being placed in a 4 °C Oxymax-CLAMS-HC system chamber, the initial rectal temperature of mice was measured. A probe was used to measure rectal temperature at 0, 3, and 6 h, while the mice remained in the cold chamber with free access to food and water.

### 4.7. Quantification PCR

Total RNA was extracted from IWAT and the liver using TRIzol^®^ (Invitrogen, Waltham, MA, USA). First-strand cDNA was synthesized using a Maxima First Strand cDNA Synthesis Kit. Real-time PCR was performed using PowerUp SYBR™ green master mix on a QuantStudio™ 3 system (Applied Biosystems, Waltham, MA, USA) to measure target gene expression. Relative gene expression was calculated using the 2^−ΔΔCt^ method normalized to 36B4 or β-actin housekeeping genes. Primer sequences of target genes are listed in Table S2.

### 4.8. Hematoxylin and Eosin (H&E) Staining

Furthermore, 5 μm-thick paraffin-embedded IWAT sections underwent deparaffinization, rehydration with xylene and ethanol, and washing. Sections were stained with hematoxylin, washed, stained with eosin, dehydrated, cleared in xylene, and mounted with xylene-based medium. Tissue processing, sectioning, and H&E staining were performed by the Mayo Clinic histology core, and slides were evaluated by a certified pathologist.

### 4.9. Adipocyte Size

Adipocyte size quantification was performed by capturing images of H&E-stained histological sections of IWAT from mice using the Cytation 5 Cell Imaging Multimode Reader (Agilent Technologies, Inc., Santa Clara, CA, USA). Image J software (version 1.51) was used for the fat cell area measurements.

### 4.10. Immunohistochemistry

Formalin-fixed IWAT and liver samples underwent immunohistochemical staining using the Vectastain ABC kit. Subsequently, the sections were counterstained with hematoxylin. The slides were captured and visualized using the EVOS^®^ autofluorescence microscope (Echo, Inc., San Diego, CA, USA). The quantification of 3,3′-Diaminobenzidine (DAB; Chromogen) on slides with hematoxylin was performed following a previously established protocol by Nguyen DH et al. [33].

### 4.11. Serum Glucose, Insulin, Leptin, and Lipid Profile Analysis

Serum glucose, cholesterol, and triglycerides were analyzed using the Heska Model 7000 Dri-Chem Veterinary Chemistry Analyzer at the University of Arizona’s Animal Care Department. Insulin and leptin levels were measured using ELISA kits from Invitrogen.

### 4.12. LC-MS/MS Measurement of FSK

#### 4.12.1. Serum Preparation

A total of 50 μL of each serum sample was combined with 550 μL of methanol for protein precipitation and metabolite extraction. After vortexing and storage at −20 °C, the mixture was centrifuged at 2400× *g* for 10 min at 4 °C. The supernatants (450 μL) were collected and dried with a CentriVap Concentrator. The dried samples were reconstituted in 150 μL of methanol. A pooled sample was used as the quality control (QC) sample.

#### 4.12.2. Tissue Preparation

A Bullet Blender homogenizer (Next Advance, Averill Park, NY, USA) was used to homogenize each tissue sample (~20 mg) in 200 µL of methanol/PBS (4:1, *v*:*v*). After adding 800 µL of methanol/PBS (4:1, *v*:*v*) and vortexing, the samples were frozen, followed by sonication in an ice bath. The supernatant from centrifugation was transferred to a new tube and dried under vacuum. The residue was reconstituted in 150 µL methanol for MS analysis, while a pooled QC sample was also prepared.

#### 4.12.3. Targeted LC-MS/MS

An Agilent 1290 UPLC-6495 was used for all mass spectrometry experiments [34,35,36]. Each sample was analyzed in negative ion mode using 6 μL per injection. Reverse-phase chromatography was performed using a Waters XSelect HSS T3 column (150 × 2.1 mm, 2.5 µm particle size; Waters Corporation, Milford, MA, USA) with a 0.3 mL/min flow rate. Solvent A was 10 mM ammonium acetate in 60% H_2_O/40% acetonitrile, and Solvent B was 10 mM ammonium acetate in 90% isopropyl alcohol/10% acetonitrile. Isocratic elution was performed with 50% solvent B for 3 min, followed by a 12 min progressive rise to 100% B. At t = 25 min, the percentage of solvent B was gradually reduced to 50% to prepare for the next sample injection after 10 min of continuous 100% solvent B. The LC-MS/MS system was optimized with the FSK chemical standard and controlled by the Agilent Masshunter Workstation software (version 12.1).

### 4.13. Safety Evaluation

To evaluate the safety of FSK and Tween80 treatments, serum concentrations of alkaline phosphatase (ALP), alanine transaminase (ALT), creatine phosphokinase (CPK), and aspartate transaminase (AST) were measured as demonstrated in Section 4.11.

### 4.14. Statistical Analyses

Statistical software “SPSS_25_” was utilized for data analysis. Data were assessed for normality, and log transformation was applied when necessary to achieve a normal distribution. A one-way ANOVA followed by a Tukey HSD Post Hoc test was employed to assess the means of multiple groups. A two-tailed Student’s t-test was utilized to compare the means of the two groups. The data are presented as the mean ± standard error of the mean. Statistical significance was determined at a *p*-value of less than 0.05.

## 5. Conclusions

FSK administration in obese mice did not result in significant reductions in body weight or fat mass, but was associated with trends toward decreased adiposity and a reduction in adipocyte size within IWAT. Importantly, FSK-treated mice displayed im-proved glucose tolerance, lower serum insulin, and reduced total cholesterol and inflammation compared to the control group. Additionally, FSK treatment led to increased expression of genes related to thermogenesis and beige adipocyte markers in IWAT, with notably higher levels of Ucp1 and Tmem26 mRNA, as well as elevated Ucp1 protein expression. These findings suggest that FSK may promote beneficial metabolic effects, although its impact on overall body weight remains uncertain. Further research is needed to understand its full metabolic benefits and mechanism of action.

## Data Availability

The data supporting this study’s findings are available from the corresponding author upon reasonable request.

## Supplementary Materials

The following supporting information can be downloaded at https://www.mdpi.com/article/10.3390/ijms26146607/s1.

## Abbreviations

The following abbreviations are used in this manuscript:

**Abbreviation**	**Full Name**
DAB	3,3′-Diaminobenzidine
HMG-CoA reductase	3-hydroxy-3-methyl-glutaryl-coenzyme A reductase
Acc1	Acetyl-CoA Carboxylase 1
AC	Adenylate cyclase
ATGL	Adipose triglyceride lipase
ALP	Alkaline phosphatase
ALT	Alanine transaminase
AST	Aspartate transaminase
BW	Body weight
Cpt1	Carnitine palmitoyl transferase I
Cidea	Cell death-inducing DFFA-like effector a
CFE	Coleus forskohlii root extract
CREB	cAMP-responsive transcription factors
CPK	Creatine phosphokinase
cAMP	Cyclic adenosine monophosphate
F4/80	EGF-like module-containing mucin-like hormone receptor-like 1
Fabp1	Fatty Acid-Binding Protein 1
FSK	Forskolin
FSK15^both^	FSK 15 mg/kg Body Weight/injection into both inguinal WAT depots
FSK7.5^both^	FSK 7.5 mg/kg Body Weight/injection into both inguinal WAT depots
FSK7.5^left^	FSK 7.5 mg/kg Body Weight/injection into the left inguinal WAT depot
GTT	Glucose tolerance test
Glut2	Glucose transporter 2
Glut4	Glucose transporter 4
G6P	Glucose 6 phosphatase
GWAT	Gonadal WAT
H&E	Hematoxylin and eosin
HFD	High-fat diet
HSL	Hormone-sensitive lipase
Il6	Interleukin-6
Mcp1	Monocyte chemoattractant protein
MAPK	P38 mitogen-activated protein kinase
Pgc1α	Peroxisome proliferator-activated receptor γ co-activator1α
PEPCK	Phosphoenolpyruvate carboxykinase
PKA	Protein kinase A
QC	Quality control
RER	Respiratory exchange ratio
Serbp1c	Sterol regulatory element-binding protein-1c
Scd1	Stearoyl-CoA desaturase 1
Tmem26	Transmembrane protein 26
Tnfα	Tumor necrosis factor α
Con^both^	Tween 80 Control into both IWAT depots
Ucp1	Uncoupling protein 1
WAT	White adipose tissue
