# Supplementary material for: Anti-Obesity and Metabolic Effects of Forskolin in Obese C57BL/6J Mice
Source: Int J Mol Sci. 2025 Jul 10;26(14):6607. doi: 10.3390/ijms26146607 (PMC12295497; doi:10.3390/ijms26146607)
Supplement: Supplementary file 1 [file ijms-26-06607-s001.zip › ijms-3609927-supplementary.pdf]

Table S1. Serum chemistry panel for safety evaluation of FSK and Tween80

		FSK15^{both}	FSK7.5^{both}	FSK7.5^{left}	Con^{both}
		Renal Panel			
**Total Protein**	**g/dL**	4.18 ± 0.07	4.44 ± 0.05	4.56 ± 0.02	4.55 ± 0.12
**Albumin**	**g/dL**	1.92 ± 0.02	2.04 ± 0.02	2.04 ± 0.02	2.06 ± 0.07
**Creatinine**	**mg/dL**	0.18 ± 0.02	0.20 ± 0.00	0.22 ± 0.02	0.20 ± 0.00
**BUN**	**mg/dL**	24.54 ± 1.66	28.80 ± 0.94	29.2 ± 0.92	28.10 ± 2.26
		Liver Panel			
**Total bilirubin**	**mg/dL**	0.30 ± 0.03	0.30 ± 0.06	0.30 ± 0.06	0.32 ± 0.12
**ALP**	**U/L**	58.20 ± 4.43	61.20 ± 2.48	62.80 ± 5.13	46.20 ± 4.49
**ALT**	**U/L**	22.20 ± 2.22	21.80 ± 1.24	31.00 ± 6.19	23.25 ± 5.79
**AST**	**U/L**	46.40 ± 2.48	51.00 ± 3.39	58.40 ± 5.15	74.00 ± 15.40
		Electrolytes Panel			
**Calcium**	**mg/dL**	8.74 ± 0.12	8.94 ± 0.12	9.12 ± 0.11	8.94 ± 0.02
**Phosphorus**	**mg/dL**	7.10 ± 0.34	6.28 ± 0.21	6.62 ± 0.31	6.33 ± 0.25
		Other			
**CPK**	**U/L**	86.80 ± 10.45	90.80 ± 23.36	81.40 ± 21.59	117.20 ± 18.85
**Globulin**	**g/dL**	2.26 ± 0.07	2.40 ± 0.03	2.52 ± 0.05	2.50 ± 0.12

Table S2. Primer sequences

**36B4**	Forward	GCTTCGTGTTCACCAAGGAGGA
	Reverse	GTCCTAGACCAGTGTTCTGAGC
**β-actin**	Forward	GTGACGTTGACATCCGTAAAGA
	Reverse	GCCGGACTCATCGTACTCC
**UCP1**	Forward	GCTTTGCCTCACTCAGGATTGG
	Reverse	CCAATGAACACTGCCACACCTC
**PGC-1α**	Forward	GAATCAAGCCACTACAGACACCG
	Reverse	CATCCCTCTTGAGCCTTTCGTG
**PPARγ**	Forward	GTACTGTCGGTTTCAGAAGTGCC
	Reverse	ATCTCCGCCAACAGCTTCTCCT
**PRDM16**	Forward	ATCCACAGCACGGTGAAGCCAT
	Reverse	ACATCTGCCCACAGTCCTTGCA
**IL6**	Forward	GTTCTCTGGAAATCGTGGA
	Reverse	GCCACTCCTTCTGTGACTCC
**TMEM26**	Forward	ACCCTGTCATCCCACAGAG
	Reverse	TGTTTGGTGGAGTCCTAAGGTC
**ELOVL3**	Forward	ACAGAGGCACACACAAACAC
	Reverse	GATAGGGAAGCAGGGTCTCC
**Leptin**	Forward	TGGGGTTTTGGAGCAGTTTG
	Reverse	CTGTCACTCTTTCCCGGTCT
**CIDEA**	Forward	GGTGGACACAGAGGAGTTCTTTC
	Reverse	CGAAGGTGACTCTGGCTATCC
**MCP1**	Forward	TCGCTCAGCCAGATGCAAT
	Reverse	ATCTCCTTGGCCACAATGGTC
**GLUT2**	Forward	TGAGTTCCTTCCAGTTCGGC
	Reverse	CTGAGGCCAGCAATTCTGACT
**PI3K**	Forward	ACACCAACGGTTTGGACTATGG
	Reverse	GGCTACAGTTAGTGGGCTTGG
**NRF1**	Forward	GCAGCACCTTTGGAGAATGTG
	Reverse	AATTAACCTCCTGTGGCGCAG
**GLUT4**	Forward	GCTCTGACGTAAGGATGGGG
	Reverse	TGGCCAGTTGGTTGAGTGTT
**PEPCK**	Forward	AATGCTTTCTCAAAGTCCTC
	Reverse	AATATGACCAACTGTTGGCTG
**G6P**	Forward	AGATAGCAAGAGTAGAAGTGAC
	Reverse	TTCCAAGTGGATTCTGTTTGG
**TNFα**	Forward	CCTGTAGCCCACGTCGTAG
	Reverse	GGGAGTAGACAAGGTACAACCC
**Ac1**	Forward	CCTTCTCCAACGTGATGACCTG
	Reverse	GCTGATGTACCTGTTGACGCGT
**Ac2**	Forward	GATTGGTCTCCAGAAGTGGCAG
	Reverse	CCATTCAAGTGCTCCAGAGTGAC
**Ac3**	Forward	CCTCTGAGGATGAGCACGAACT
	Reverse	GAGTAGCGTGTTTCCATCTCTGG

**Ac4**	Forward	CAACCTCCTGACGCTGTACTTC
	Reverse	CCTGGTTGTCACCAGCATTTGG
**Ac5**	Forward	TCGCAATGCCTACCTCAAGGAG
	Reverse	GCGGATTGTGTCCGATGGAGTT
**Ac6**	Forward	TAGATCCTCGCTTCGGAGCCTA
	Reverse	CAACAGCAGGAAGATAGCGGCA
**Ac7**	Forward	GACGAGATGCTGTCAGCCATTG
	Reverse	CACGCTCAAAGCCCTTCTCCAA
**Ac8**	Forward	CTGCTCACAGAGACCATCTACG
	Reverse	CAGCAGTGATGCTTCCTTGGTC
**Ac9**	Forward	GCAAAATGGCTGTCAAGACGAGC
	Reverse	CTGGCTGTTAGTGAGCTTCTCC
**Ac10**	Forward	CTTGTGAGTGGTGTCAGGCTGA
	Reverse	CAAACAGCAGCTCCGTGGTGAA
